# Cyanidin-3-*O*-glucoside protects the brain and improves cognitive function in APPswe/PS1ΔE9 transgenic mice model

**DOI:** 10.1186/s12974-023-02950-3

**Published:** 2023-11-17

**Authors:** Hana Baek, Sanjay, Miey Park, Hae-Jeung Lee

**Affiliations:** 1 Department of Food Science and Biotechnology, College of BioNano Technology, Gachon University, Seongnam-si, Gyeonggi-do 13120 Republic of Korea; 2 Department of Food and Nutrition, College of BioNano Technology, Gachon University, Seongnam-si, Gyeonggi-do 13120 Republic of Korea; 3 Institute for Aging and Clinical Nutrition Research, Gachon University, Seongnam-si, Gyeonggi-do 13120 Republic of Korea; 4 Department of Health Sciences and Technology, GAIHST, Gachon University, Incheon, 21999 Republic of Korea

**Keywords:** Cyaninidin-3-O-glucoside, Anthocyanins, Alzheimer’s disease, Autophagy, Neuronal apoptosis, Tau pathology, Synaptic plasticity

## Abstract

Cyanidin-3-O-glucoside (C3G) is a natural anthocyanin with antioxidant, anti-inflammatory, and antitumor properties. However, as the effects of C3G on the amyloidogenic pathway, autophagy, tau phosphorylation, neuronal cell death, and synaptic plasticity in Alzheimer’s disease models have not been reported, we attempted to investigate the same in the brains of APPswe/PS1ΔE9 mice were analyzed. After oral administration of C3G (30 mg/kg/day) for 16 weeks, the cortical and hippocampal regions in the brains of APPswe/PS1ΔE9 mice were analyzed. C3G treatment reduced the levels of soluble and insoluble Aβ (Aβ40 and Aβ42) peptides and reduced the protein expression of the amyloid precursor protein, presenilin-1, and β-secretase in the cortical and hippocampal regions. And C3G treatment upregulated the expression of autophagy-related markers, LC3B-II, LAMP-1, TFEB, and PPAR-α and downregulated that of SQSTM1/p62, improving the autophagy of Aβ plaques and neurofibrillary tangles. In addition, C3G increased the protein expression of phosphorylated-AMPK/AMPK and Sirtuin 1 and decreased that of mitogen-activated protein kinases, such as phosphorylated-Akt/Akt and phosphorylated-ERK/ERK, thus demonstrating its neuroprotective effects. Furthermore, C3G regulated the PI3K/Akt/GSK3β signaling by upregulating phosphorylated-Akt/Akt and phosphorylated-GSK3β/GSK3β expression. C3G administration mitigated tau phosphorylation and improved synaptic function and plasticity by upregulating the expression of synapse-associated proteins synaptophysin and postsynaptic density protein-95. Although the potential of C3G in the APPswe/PS1ΔE9 mouse models has not yet been reported, oral administration of the C3G is shown to protect the brain and improve cognitive behavior.

**Graphical Abstract:**

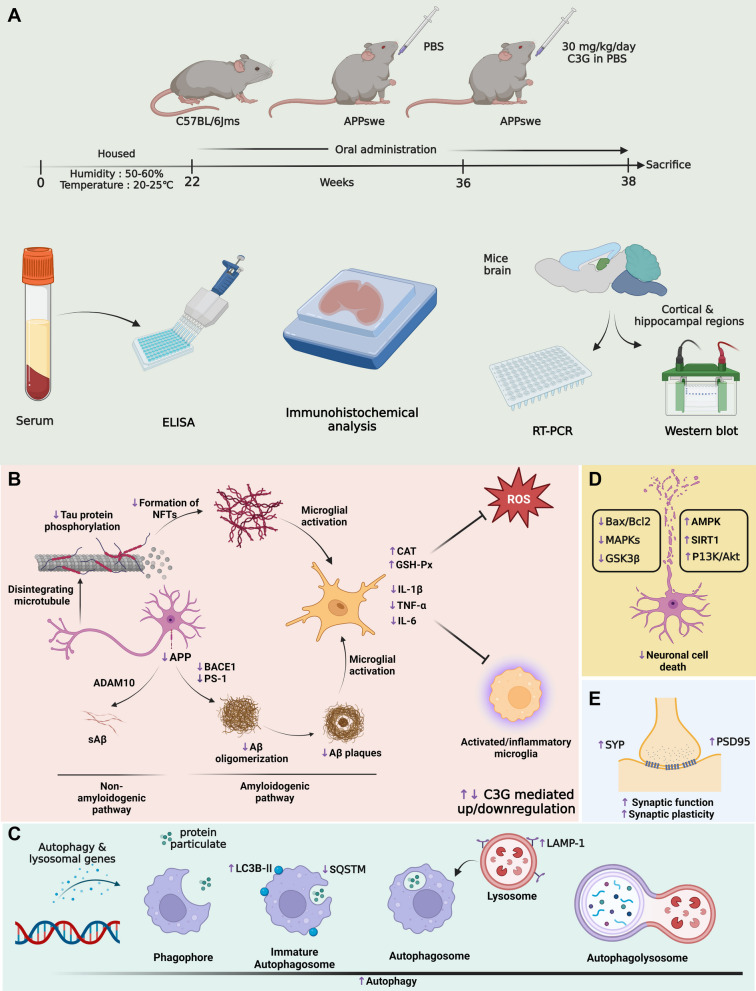

## Introduction

Alzheimer’s disease (AD) is the most common cause of dementia, affecting approximately 50 million people worldwide. This number is expected to double in 5 years, with an estimated 152 million cases by 2050 [1, 2]. AD is considered an aging disease as it is common in patients aged 65 years or older. It is characterized by the impairment of an individual’s ability to think clearly, difficulty performing basic day-to-day tasks, confusion, and poor memory and judgment [3, 4]. Although AD pathology remains unelucidated, AD progression has been attributed to the presence of β-amyloid (Aβ) plaques and neurofibrillary tangles (NFTs) within the brain.

The amyloid precursor protein (APP) is a transmembrane glycoprotein expressed in several cells, including central nervous system neurons. APP is processed via the non-amyloidogenic and amyloidogenic pathways. In a non-amyloidogenic way, APP is catalyzed by α-secretase (ADAM10; A disintegrin and metalloproteinase domain-containing protein 10) and λ-secretase to produce soluble Aβ fragments. In contrast, in the amyloidogenic pathway, APP is catalyzed by β-secretase (BACE1; beta-site APP cleaving enzyme 1) to produce insoluble peptides [5]. The Aβ40 and Aβ42 isoforms are the major cytotoxic peptides associated with neurodegeneration [6]. The microtubular protein tau is also essential in providing internal support and carrying nutrients to neurons. However, in AD, it becomes hyper phosphorylated and disorganized to form NFTs [4, 7, 8].

In autophagy, macromolecular aggregates, damaged organelles, and long-lived proteins are cleared from the cell via lysosomal degradation, essential for protein homeostasis in the central nervous system [9, 10]. Impaired autophagy is strongly associated with AD pathology [11–15]. In AD, the expression of several core autophagy-related genes, such as microtubule-associated protein 1A/1B-light chain 3 (LC3B-II) and lysosomal-associated membrane protein 1 (LAMP-1), is downregulated in the brain [16–19]. Transcription factor EB (TFEB), a master transcriptional regulator of autophagy, enhances gene expression that controls autophagosome formation, lysosome function, and autophagic flux. TFEB dysfunction is strongly associated with AD pathogenesis [9], and the disruption of autophagic flux is indicated by the accumulation of sequestosome 1 (SQSTM1/p62) [20].

Neuronal cell death is the primary concern in neurodegenerative diseases (NDs). The ratio of the apoptosis regulator, B-cell lymphoma 2 (Bcl-2) associated X (Bax), to Bcl-2 protein has been associated with hyperphosphorylation of tau and neuronal death in an in vivo model [21]. Additionally, Bcl-2 overexpression decreases NFTs and Aβ plaque formation in mice and ameliorates cognitive impairment [22]. Mitogen-activated protein kinases (MAPKs) are critical cellular pathways that regulate various cellular activities, including gene expression, mitosis, metabolism, apoptosis, proliferation, differentiation, and movement [23]. Activation of P38 MAPKs and extracellular signal-regulated kinases (ERK)1/2 is involved in tau hyperphosphorylation [24]. Adenosine monophosphate-activated protein kinase (AMPK) also modulates tau phosphorylation and pathology in vivo [25]. Sirtuin 1 (SIRT1), a central downstream molecule of the AMPK signaling pathway, is involved in the pathogenesis of NDs [26]. Shah et al. [27] revealed the involvement of the AMPK/SIRT1 signaling pathway in the modulation of Aβ deposition and cognitive function in AD rats [23, 24]. The phosphoinositide 3-kinase/protein kinase B (PI3K/Akt) signaling is also compromised in AD neurons [28, 29], and Aβ deposition inhibits PI3K/Akt activation, thus triggering the expression of pro-apoptotic factors, such as glycogen synthase kinase-3 beta (GSK-3β) and nuclear factor kappa-light-chain-enhancer of activated B cells (NF-κB), resulting in neuronal apoptosis [30]. Synapses are considered an early site of dysfunction/pathology in AD, and synaptic loss strongly correlates with cognitive decline [31, 32]. Animal studies suggest that synaptophysin (SYP) and postsynaptic density protein-95 (PSD95) disruption are associated with cognitive and learning deficits [33, 34]. Synapse-associated proteins, including synapsin (SYN-1), SYP, and PSD95, play crucial roles in synaptic plasticity and memory formation [35]. The reduced expression of SYP and PSD95 has been observed in the brain tissue of patients with AD [36–38].

Anthocyanins are flavonoids found in the fruits of various plants, including blackcurrants, blueberries, grapes, black beans, eggplants, and honeyberries [39]. Cyanidin-3-O-glucoside (C3G) is an anthocyanin present in various fruits and vegetables, mostly berries, blue and red fruits, and vegetables, and their bioactivity and daily intake are associated with the plant source. C3G is reported to play various bioactivities such as antioxidant, anti-inflammatory, anti-apoptotic or antimicrobial activities [40, 41]. Recent studies have reported the bio-functionality of C3G is due to its metabolites and almost 20 different types of metabolites that are found in human blood. Although the exact mechanism and function of C3G are yet to be explored, protocatechuic acid, phloroglucinaldehyde, ferulic acid, vanillic acid, and their derivatives are thought to be the primary bioactive metabolites responsible for C3G-mediated antioxidation and anti-inflammation [42]. C3G also inhibited signal transduction and neuronal cell death by inhibiting mitochondrial depolarization and reactive oxygen species formation in glutamate-induced rat hippocampal neurons [43]. Also, C3G has been reported to cross the blood–brain barrier, making it a suitable candidate for neurodegenerative studies [44]. We previously reported the in vivo immunomodulatory and antioxidant properties of C3G through transcriptomic analysis and its effects on microglial polarization in an in vitro AD model [45, 46]. Although various studies have demonstrated the role of C3G in improving cognitive impairment and AD [47–49], the effects of C3G on autophagy, tau protein phosphorylation, neuronal cell death, and synaptic plasticity remain unexplored. Herein, we assessed the same in an APPswe/PS1ΔE9 transgenic mouse model.

## Materials and methods

### Materials

C3G was purchased from ChemFaces Biochemical Co. (Wuhan, China). Antibodies against LC3B (ab48394), SQSTM1 (ab91526), TFEB (ab245350), LAMP-1 (ab24170), Bcl-2 (ab182858), Bax (ab32503), PPARα (ab24509), AMPK (ab3759), p-AMPK (T183) (ab133448), β-actin (ab6276), SIRT1 (ab110304), PS-1 (ab15458), APP (ab32136), BACE1 (ab183612), and ADAM10 (ab124695) were purchased from Abcam (Cambridge, UK). Antibodies against PSD95 (3450S), HO-1 (43966S), Tau (46687S), Phospho-Tau (Thr205) (49561S), Phospho-p38 MAPK (Thr180/Tyr182) (4511S), p38 MAPK (8690S), Phospho-p44/42 MAPK (Thr202/Tyr204) (4370S), p44/42 MAPK (4695S), GSK-3β (12456S), Phospho-GSK-3β (Ser9) (5558S), Akt (9272S), and Phospho-Akt (Ser473) (4051S) were obtained from Cell Signaling Technology (Danvers, MA, USA). Anti-SYP antibody (MA5-14532) was purchased from Invitrogen (Waltham, MA, USA).

### Animals and diets

**Scheme 1:**
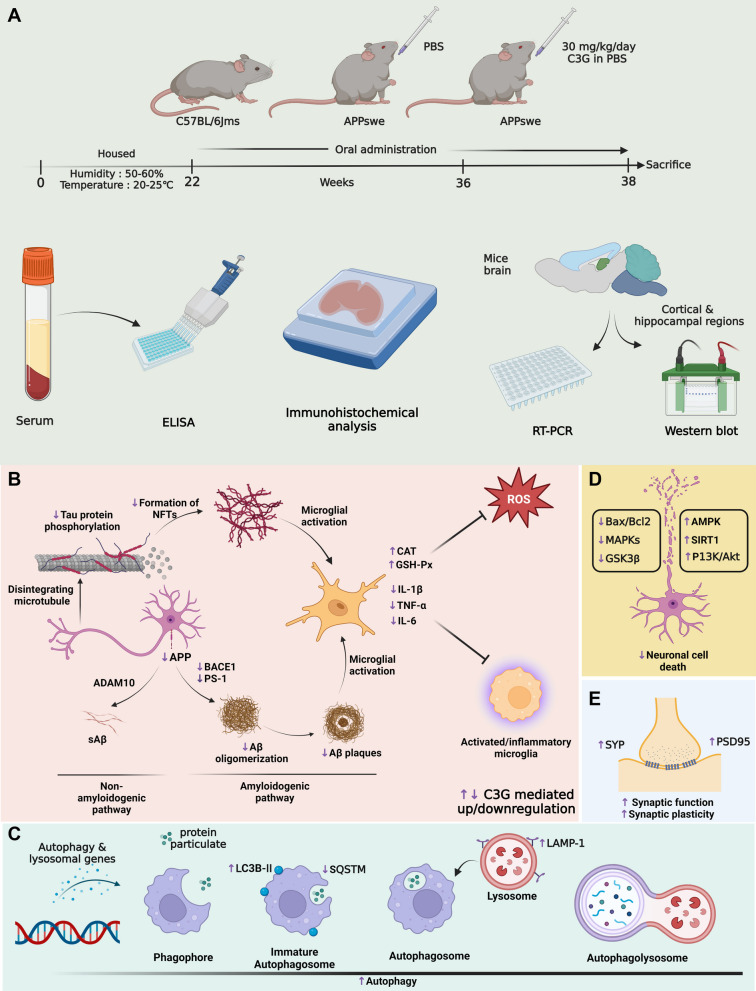
Experimental procedure and targeted molecular signaling. **A** Experimental protocol; **B** C3G-mediated amyloidogenic pathway and tau protein phosphorylation; **C** C3G enhanced autophagy in APPswe mice; **D** neuroprotective effects of C3G on neuronal apoptosis; and **E** C3G improved the synaptic function. APP, amyloid precursor protein; BACE1, β-secretase; PS-1, presenilin-1; ADAM10, α-secretase; Aβ, amyloid beta; IL, interleukin; TNF-α, tumor necrosis factor-α; GSH-Px, glutathione peroxidase; CAT, catalase; NFTs, neurofibrillary tangles; C3G, cyaninidin-3-O-glucoside; Bax, Bcl-2 associated X, apoptosis regulator; Bcl2, B-cell lymphoma 2; MAPKs, mitogen-activated protein kinases; GSK3β, glycogen synthase kinase-3 beta; AMPK, AMP-activated protein kinase; SIRT1, sirtuin 1; PI3K/Akt, phosphoinositide 3-kinase/protein kinase B; SYP, synaptophysin; PSD95, postsynaptic density protein-95; LC3B-II, light chain 3BII; LAMP-1, lysosomal-associated membrane protein 1; TFEB, transcription factor EB; SQSTM, sequestosome 1.

Nine-week-old female heterozygous AD mice B6C3-Tg (APPswe, PSEN1ΔE9)85Dbo/Mmjax were acquired from Jackson Laboratory (Sacramento, CA, USA). Non-transgenic mice (C57BL/6Jms) of the same age were obtained from SLC (Hamamatsu City, Shizuoka Prefecture, Japan) and used as the negative control. All mice were housed at 20–25 °C temperature at 50–60% humidity with a 12 h light/dark cycle for two months. After acclimatization, the mice were randomly divided into three groups (n = 8): non-transgenic mice (NC), AD mice orally administered PBS (APPswe), and AD mice orally administered 30 mg/kg/day C3G in PBS (APPswe_C3G) for 16 weeks which was adjusted according to a previous study [50], where 50 mg/kg/body weight of C3G for 8 weeks was given. As we planned to administer orally to AD mice for 16 weeks, we modified C3G’s dose to 30 mg/kg/day. The mice were cared for 38 weeks with free access to food and water (Scheme 1A). Food intake and body weight were measured twice and once a week, respectively. All experiments using the mouse model were approved by Eulji University (EUIACUC20-13) and performed according to the Guide for the Care and Use of Laboratory Animals by the Ministry of Food and Drug Safety.

### Immunohistochemical thioflavin-S staining

After 38 weeks, mice were sacrificed by CO_2_ asphyxiation after 12 h of fasting. Blood was collected by cardiac puncture, and the brains were removed. The cortex and hippocampus were separated. Three mice per group were euthanized, and perfusion was performed using a gravity-fed system through the left ventricle. The left ventricle was first perfused at a pressure of 100 cm and a rate of 10 ml/min rate using 50 ml of 4% paraformaldehyde [51]. Approximately 10 min after perfusion, the brains were removed and fixed in 10% formalin. Thioflavin-S staining [52] was performed for immunotherapy targeting the amyloid peptides in the brain tissue. The hippocampal and cortical regions of coronal brain sections were analyzed using ImageJ (U. S. National Institutes of Health, Bethesda, Maryland, USA) to determine the number of Aβ plaques [53].

### Enzyme-linked immunosorbent assay (ELISA)

The brain tissue samples were homogenized in ice-cold 20 mM Tris [pH 8.5 (soluble)] or 5 M guanidine HCl/50 mM Tris–HCl [pH 8.0 (insoluble)] to determine the Aβ1-40 or Aβ1-42 levels. The amounts of soluble and insoluble Aβ in the serum were analyzed using Aβ1–40 and Aβ1–42 ELISA kits (Invitrogen, Waltham, MA, USA). The levels of tumor necrosis factor-α (TNF-α), interleukin (IL)-6, glutathione peroxidase (GSH-Px), and catalase (CAT) were measured in serum samples using TNF-α, IL-6 (R&D Systems, Minneapolis, USA), GSH-Px, and CAT (BlueGene Biotech, Shanghai, China) ELISA kits, respectively. The assay results were analyzed as per the manufacturer’s instructions.

### Y-maze test

The Y-maze test was performed after the oral administration of C3G for 14 weeks. The Y-maze consists of three identical arms (30 cm long, 5 cm wide, and 12 cm high) at an angle of 120° and can be used to evaluate the spatial working memory of mice [54]. At the beginning of the experiment, mice were tested without previous exposure or habit of the maze by gently placing them at the end of one arm and allowing them to explore the maze freely for 5 min. Spontaneous alternation was defined as entering three different arms during a continuous choice. The alternation ratio was calculated as the ratio of the actual number of alternations to the maximum number.

### Western blotting

For protein expression analysis, total proteins were harvested from the hippocampus and cortex (from 5 mice per group) using a lysis buffer (iNtRON Biotechnology, Gyeonggi-do, Korea) containing protease and phosphate inhibitors. The extracted proteins (30 μg) were separated on a sodium dodecyl sulfate–polyacrylamide gel and transferred onto a polyvinylidene fluoride membrane (Bio-Rad Laboratories, Hercules, CA, USA). After blocking with 5% skim milk at room temperature (RT), the membranes were incubated for 2 h at RT with the following primary antibodies: anti-LC3B (1:1000 dilution), anti-SQSTM1/p62 (1:1000 dilution), anti-TFEB (1:1000 dilution), anti-LAMP-1 (1:1000 dilution), anti-Bcl-2 (1:1000 dilution), anti-Bax (1:1000 dilution), anti-PPARα (1:200 dilution), anti-AMPK (1:1000 dilution), anti-Phospho-AMPK (1:1000 dilution), anti-p38 MAPK (1:1000 dilution), anti-Phospho-p38 MAPK (1:1000 dilution), anti-Phospho-p44/42 MAPK (Erk1/2) (1:1000 dilution), anti-p44/42 MAPK (Erk1/2) (1:1000 dilution), anti-Akt (1:1000 dilution), anti-Phospho-Akt (1:1000 dilution), anti-Tau (1:1000 dilution), anti-Phospho-Tau (1:1000 dilution), anti-GSK-3β (1:1000 dilution), anti-Phospho-GSK-3β (1:1000 dilution), anti-BACE1 (1:1000 dilution), anti-SYP; (1:1000 dilution), anti-PSD95 (1:1000 dilution), anti-APP (1:1000 dilution), anti-ADAM10 (1:1000 dilution), anti-presenilin 1 (PS1; 1:1000 dilution), anti-HO-1 (1:1000 dilution), anti-SIRT1 (1:1000 dilution), and anti-β-actin (1:5000). After 1 h of incubation with the secondary antibody at RT, protein bands were detected using Miracle-Star (iNtRON Biotechnology, Seongnam, Korea), and the reactive bands were imaged using a Quant LAS 500 system (GE Healthcare Bio-Sciences AB, Sweden).

### RNA preparation and real-time polymerase chain reaction analysis

Total RNA was isolated from homogenized hippocampal and cortical tissues using an RNA extraction kit (iNtRON Biotechnology, Gyeonggi-do, Korea) according to the manufacturer’s instructions. Total RNA (50 ng) was reverse transcribed to cDNA by PCR (TaKaRa Bio, Shiga, Japan). The synthesized cDNA was analyzed using the SYBR Green Master Mix (TaKaRa Bio, Shiga, Japan), and RT-PCR was performed using an ABI Quant Studio 3 PCR system (Applied Biosystems, Foster City, CA, USA). PCR amplification of genes, using the mouse hippocampus and cortical tissues, was performed using specific primers. The following primers (5′–3′) were used: TNF-α, forward: ACCGCAACAACGCCATCTAT and reverse: GTATCAGTGGGGGTCAGCAG; IL-6, forward: AGACAAAGCCAGAGTCCTTCA and reverse: GGTCCTTAGCCACTCCTTCTG; IL-1β, forward: AGACAAAGCCAGAGTCCTTCA and reverse: GGTCCTTAGCCACTCCTTCTG; and β-actin, forward: CTGTCCCTG-TATGCCTCTG and reverse: ATGTCACGCACGATTTCC. Gene expression was standardized using the mouse β-actin gene as the control.

### Statistical analysis

Data are presented as mean ± standard error (SE). Three replicates were performed for each experiment. Data were analyzed by one-way ANOVA and Tukey’s post-hoc test using GraphPad Prism 9 (GraphPad Software, San Diego, CA, USA). Differences with values of *p* < 0.05 were considered statistically significant. Probability (*p*) values are represented as *p < 0.05 for less than 0.05, **p < 0.01 for less than 0.01, ***p < 0.001 for values less than 0.001, and ****p < 0.0001 for values less than 0.0001.

## Results

### C3G enhanced the learning and memory of APPswe mice in behavioral tests

**Fig. 1:**
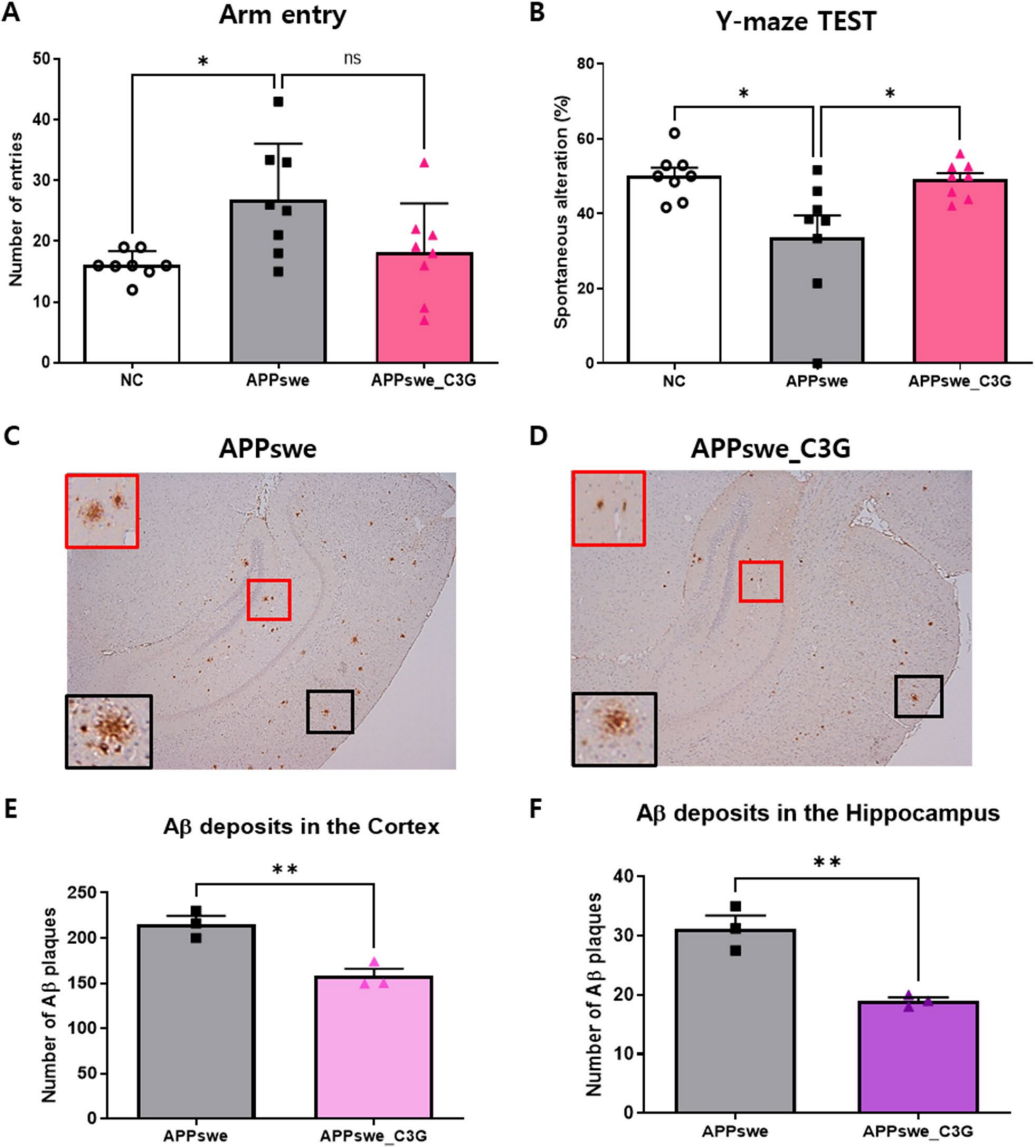
Cyanidin-3-O-glucoside (C3G) enhanced the learning and memory of APPswe mice in behavioral tests. Mice were divided into three groups: non-transgenic mice (NC), AD mice orally administered PBS (APPswe), and AD mice orally administered 30 mg/kg/day C3G in PBS (APPswe_C3G). All the mice (n = 8) underwent the Y-maze test, and the alternation ratio was calculated as the ratio of the actual number of alternations to the maximum number of alternations. The extracellular accumulation of Aβ protein was assessed using thioflavin-S staining (n = 3). Arm entry (**A**), Y-maze test (**B**), extracellular accumulation of Aβ protein through thioflavin-S staining (**C–F**). Data are representative of three independent experiments with similar results. ns, not significant; *p < 0.05, **p < 0.01. Bars represent mean ± SE

The Y-maze behavioral test measures the willingness of rodents to explore new environments. Mice with good memories are less likely to repeatedly enter the same arm. Although there was no significant difference between the APPswe and APPswe_C3G groups concerning arm entries, the spontaneous alteration rate in APPswe mice was significantly lower than that in the NC group but was increased in the APPswe_C3G group (Fig. 1A, B).

### C3G reduced the Aβ levels and deposition in APPswe mice

**Fig. 2:**
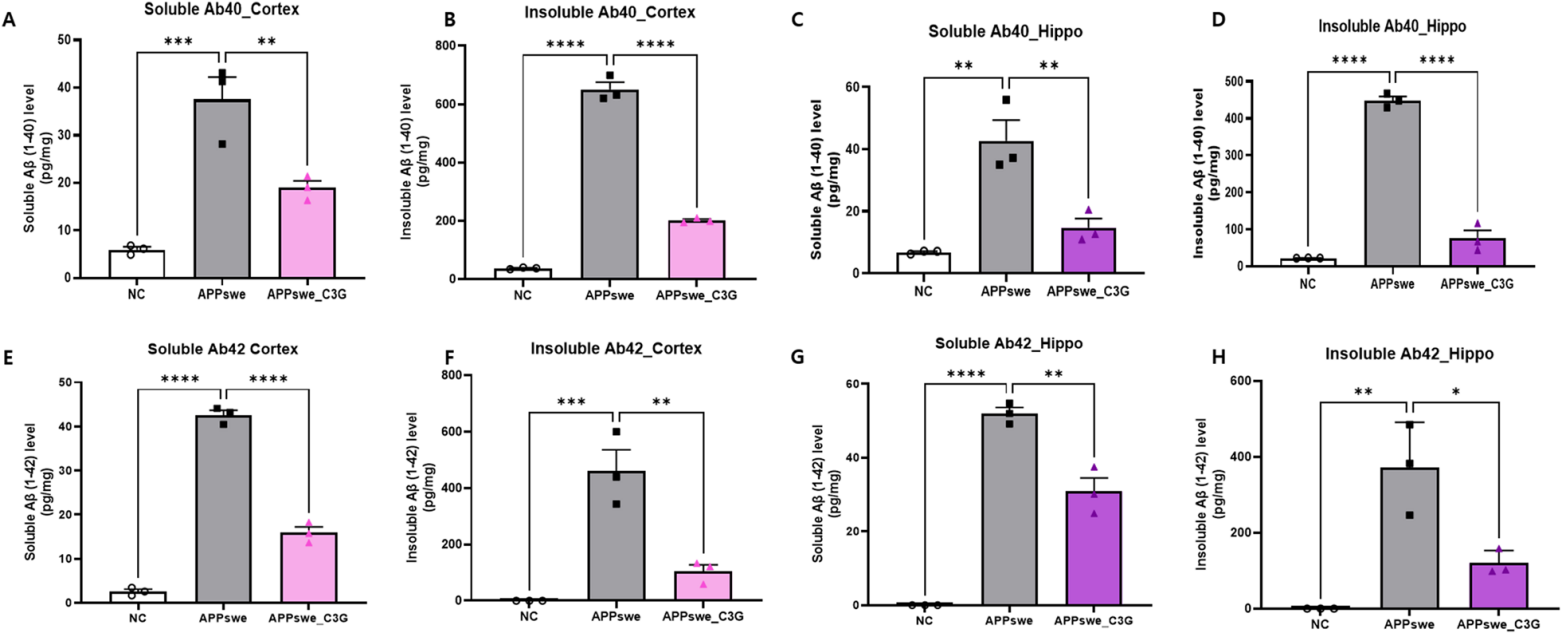
Cyanidin-3-O-glucoside (C3G) reduced Aβ levels and deposition in the cortex and hippocampus of APPswe mice. Mice were divided into three groups: non-transgenic mice (NC), AD mice orally administered PBS (APPswe), and AD mice orally administered 30 mg/kg/day C3G in PBS (APPswe_C3G). The levels of soluble and insoluble Aβ isomers (Aβ40 and A42) were observed in both the cortical and hippocampal regions of the mice brains using ELISA (n = 3). Soluble and insoluble Aβ40 in the cortex (**A, B**); soluble and insoluble Aβ1-40 in the hippocampus (**C, D**); soluble and insoluble Aβ42 in the cortex (**E, F**); and soluble and insoluble Aβ42 in the hippocampus (**G, H**). Data are representative of three independent experiments with similar results. *p < 0.05, **p < 0.01, ***p < 0.001, ****p < 0.0001. Bars represent mean ± SE

First, we confirmed the extracellular accumulation of Aβ protein using thioflavin-S staining as it is a pathological marker of AD [55]. The presence of senile plaques was observed in both the cortex (black in Fig. 1C and D) and hippocampus (red in Fig. 1C and D) of mice, which was significantly reduced in the APPswe_C3G group (Fig. 1E and F, respectively). This result demonstrated that C3G could reduce extracellular Aβ accumulation. In addition, levels of soluble and insoluble Aβ1-40 (Fig. 2A–D) and Aβ1-42 (Fig. 2E–H) peptides were measured both in the cortex and hippocampus region of the mice brains. Both soluble and insoluble Aβ1-40 and Aβ1-42 levels were significantly high in APPswe mice and significantly reduced after C3G administration, as seen in the APPswe_C3G group (Fig. 2).

### C3G enhanced autophagy in the cerebral cortical and hippocampal regions of APPswe mice

**Fig. 3:**
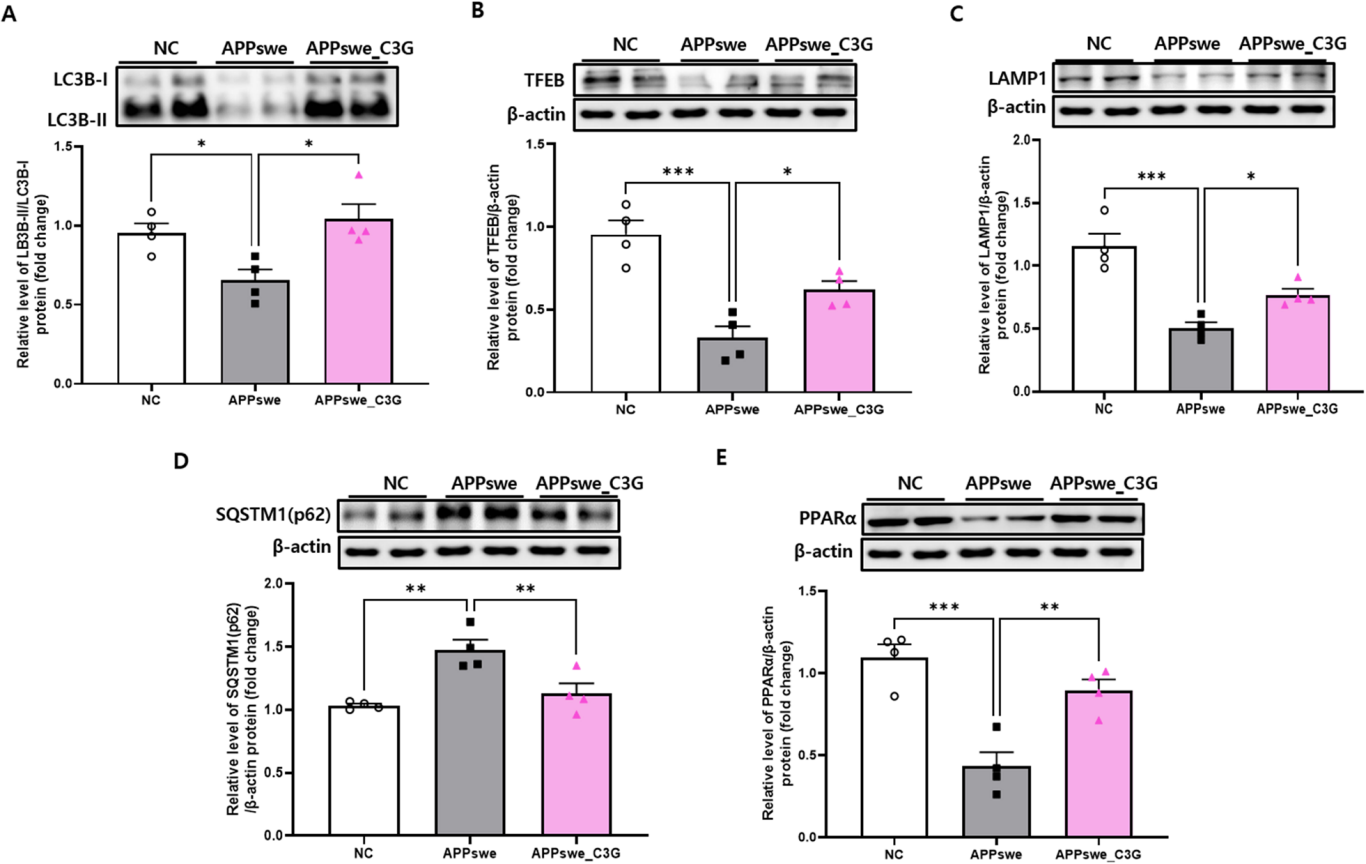
Cyanidin-3-O-glucoside (C3G) enhances autophagy in the cortex of APPswe mice. Mice were divided into three groups: non-transgenic mice (NC), AD mice orally administered PBS (APPswe), and AD mice orally administered 30 mg/kg/day C3G in PBS (APPswe_C3G). Autophagy-related markers in the cortical region of the brain were observed using western blotting (n = 4). Levels of microtubule-associated protein 1A/1B-light chain 3 (LC3B)- II/LC3B-I (**A**); transcription factor EB (TFEB) (**B**); lysosomal-associated membrane protein 1 (LAMP-1) (**C**); sequestosome 1 (SQSTM1/p62) (**D**); and peroxisome proliferator-activated receptor-α (**E**). Data are representative of three independent experiments with similar results. *p < 0.05, **p < 0.01, ***p < 0.001. Bars represent mean ± SE

**Fig. 4:**
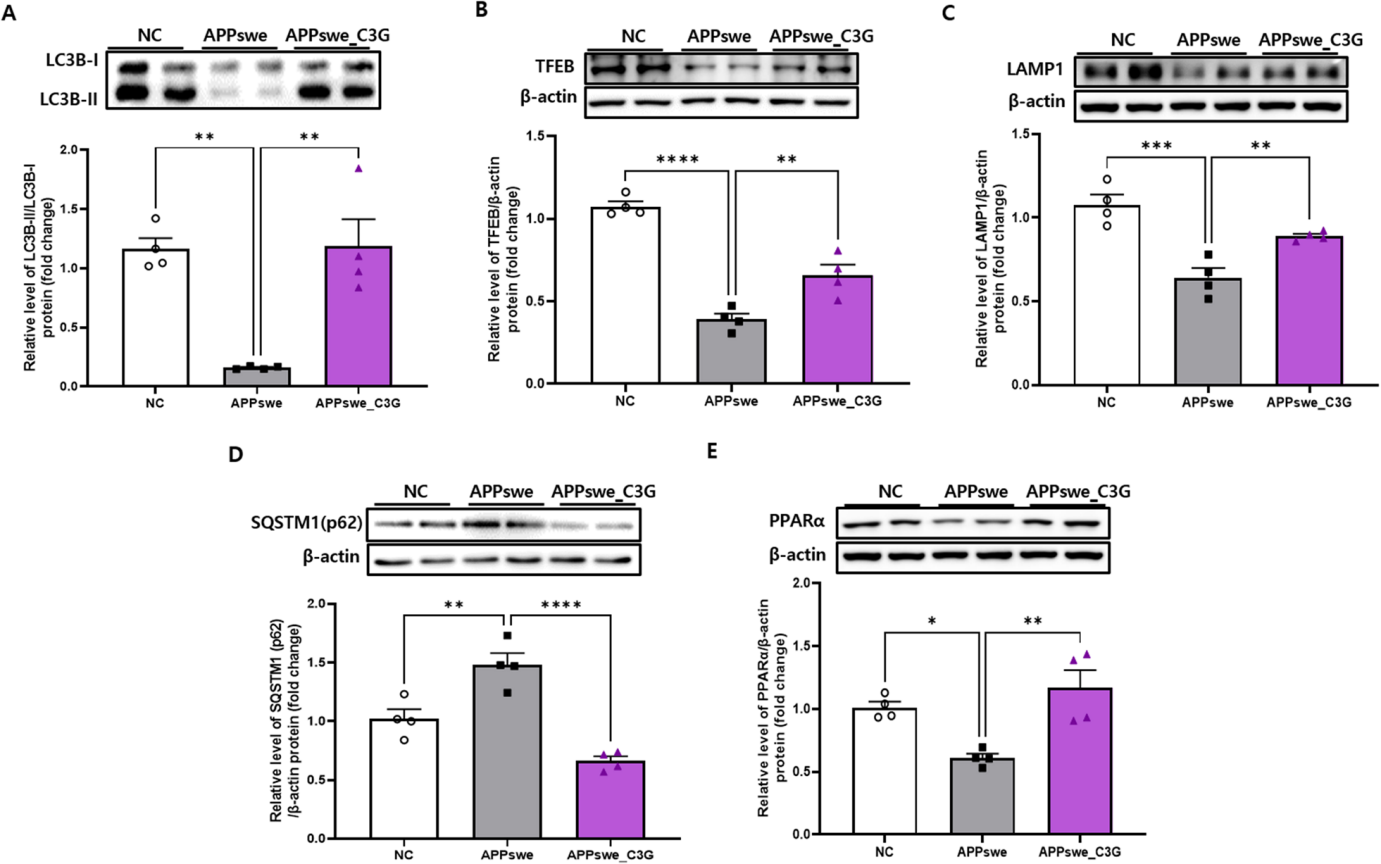
Cyanidin-3-O-glucoside (C3G) enhances autophagy in the hippocampus of APPswe mice. Mice were divided into three groups: non-transgenic mice (NC), AD mice orally administered PBS (APPswe), and AD mice orally administered 30 mg/kg/day C3G in PBS (APPswe_C3G). Autophagy-related markers in the hippocampal region of the brain were observed using western blotting (n = 4). Levels of microtubule-associated protein 1A/1B-light chain 3 (LC3)- II/LC3B-I (**A**); transcription factor EB (TFEB) (**B**); lysosomal-associated membrane protein 1 (LAMP-1) (**C**); sequestosome 1 (SQSTM1/p62) (**D**); and peroxisome proliferator-activated receptor-α (**E**). Data are representative of three independent experiments with similar results. *p < 0.05, **p < 0.01, ***p < 0.001, ****p < 0.0001. Bars represent mean ± SE

Autophagy is the primary cellular mechanism responsible for removing protein aggregates [56], and the expression of various autophagy-related factors was observed to evaluate whether C3G plays a role in inducing autophagy of Aβ plaques and NFTs in the cortex and hippocampus. The protein levels of LC3B, TFEB, and LAMP1 were significantly downregulated in APPswe mice compared to those in NC mice. However, C3G significantly increased the protein expression of LC3B (Figs. 3A and 4A), TFEB (Figs. 3B and 4B), and LAMP1 (Figs. 3C and 4C) in the APPswe_C3G group compared with that in the APPswe group (Figs. 3 and 4). Moreover, SQSTM1/p62 is considered a marker of defective autophagy, as the total cellular level of SQSTM1/p62 is inversely correlated with autophagic activity [57]. C3G significantly downregulated SQSTM1/p62 protein expression, which was considerably upregulated in the APPswe group (Figs. 3D and 4D). A recent study demonstrated that peroxisome proliferator-activated receptor- α (PPARα) activation could mediate autophagy in an in vivo murine model [58]. To confirm this hypothesis, the protein expression of PPARα has been observed in this study. We found that the protein expression of PPARα was significantly induced in the C3G-administered group compared to that in the APPswe group (Figs. 3E and 4E). These results indicate the role of C3G in inducing autophagy and that reduction in the amount of Aβ42 plaques in the APPswe_C3G group could be correlated with PPARα activation.

### C3G attenuated AD pathology

**Fig. 5:**
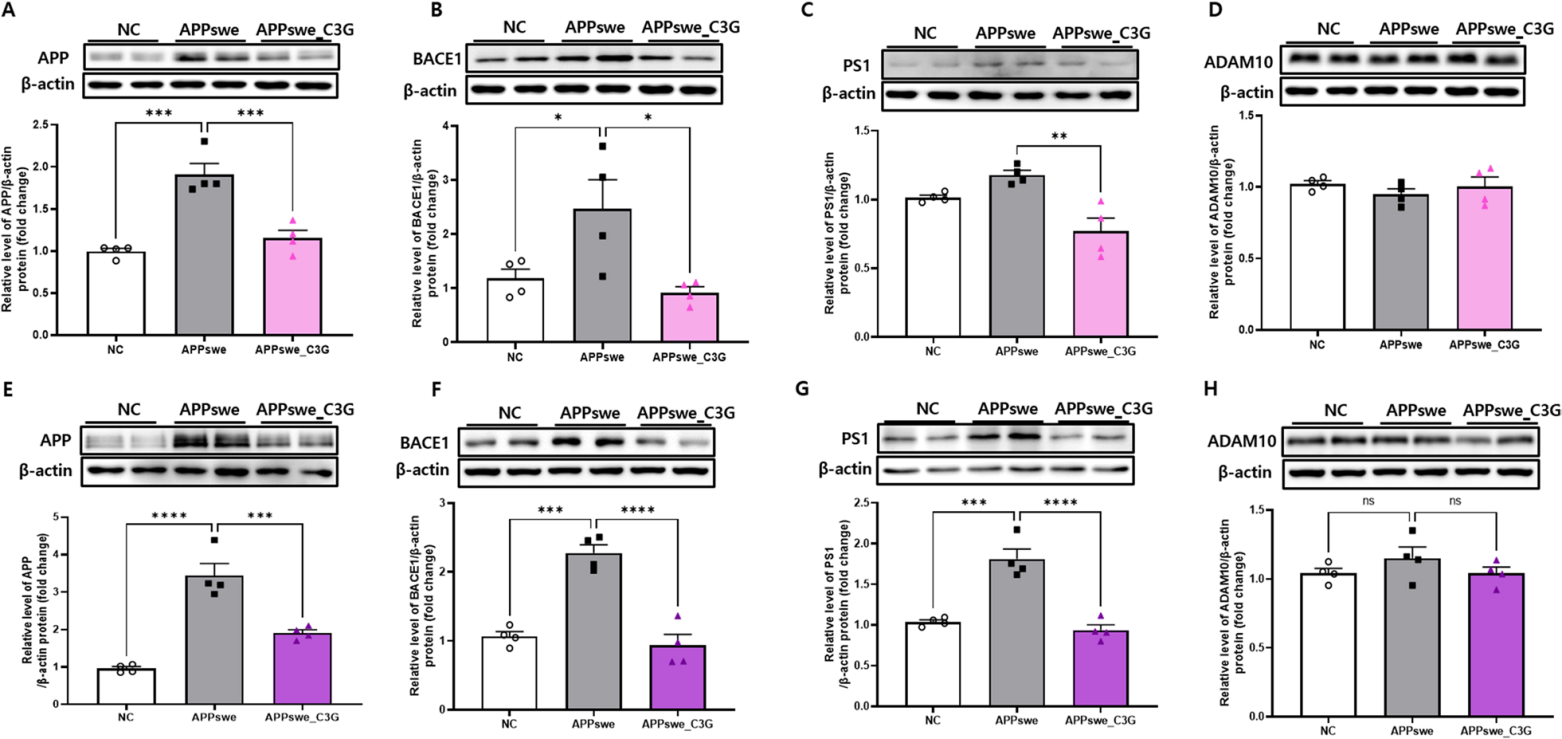
Cyanidin-3-O-glucoside (C3G) attenuated Alzheimer’s disease pathology in the cortex and hippocampus of APPswe mice. Mice were divided into three groups: non-transgenic mice (NC), AD mice orally administered PBS (APPswe), and AD mice orally administered 30 mg/kg/day C3G in PBS (APPswe_C3G). Protein expression levels in the cortical and hippocampal regions of the brain were observed using western blotting (n = 4). Levels of amyloid precursor protein (APP) (**A, E**); β-secretase (BACE1) (**B, F**); presenilin-1 (PS1) (**C, G**); and α-secretase (ADAM10) (**D, H**). Data are representative of three independent experiments with similar results. ns, not significant; *p < 0.05, **p < 0.01, ***p < 0.001, ****p < 0.0001. Bars represent mean ± SE

Mutation in PS1, a catalytic subunit of γ-secretase involved in APP processing, is one of the most common causes of AD [59]. The protein expression of APP (Fig. 5A and E), BACE1 (Figs. 5B and F), PS1 (Fig. 5C and G), and ADAM10 (Fig. 5D and H) was observed. In the cortical and hippocampal regions of APPswe mice, the protein levels of APP, BACE1, and PS1 were significantly upregulated as compared with those in the NC group, mimicking AD pathology. However, in the APPswe_C3G group, the protein expression of APP, BACE1, and PS1 was significantly downregulated compared with that in the APPswe group. Notably, the protein expression of ADAM10 was unaffected. These results demonstrated the role of C3G in attenuating the amyloidogenic pathway in AD.

### C3G regulated neuroinflammation and antioxidation in APPswe mice

**Fig. 6:**
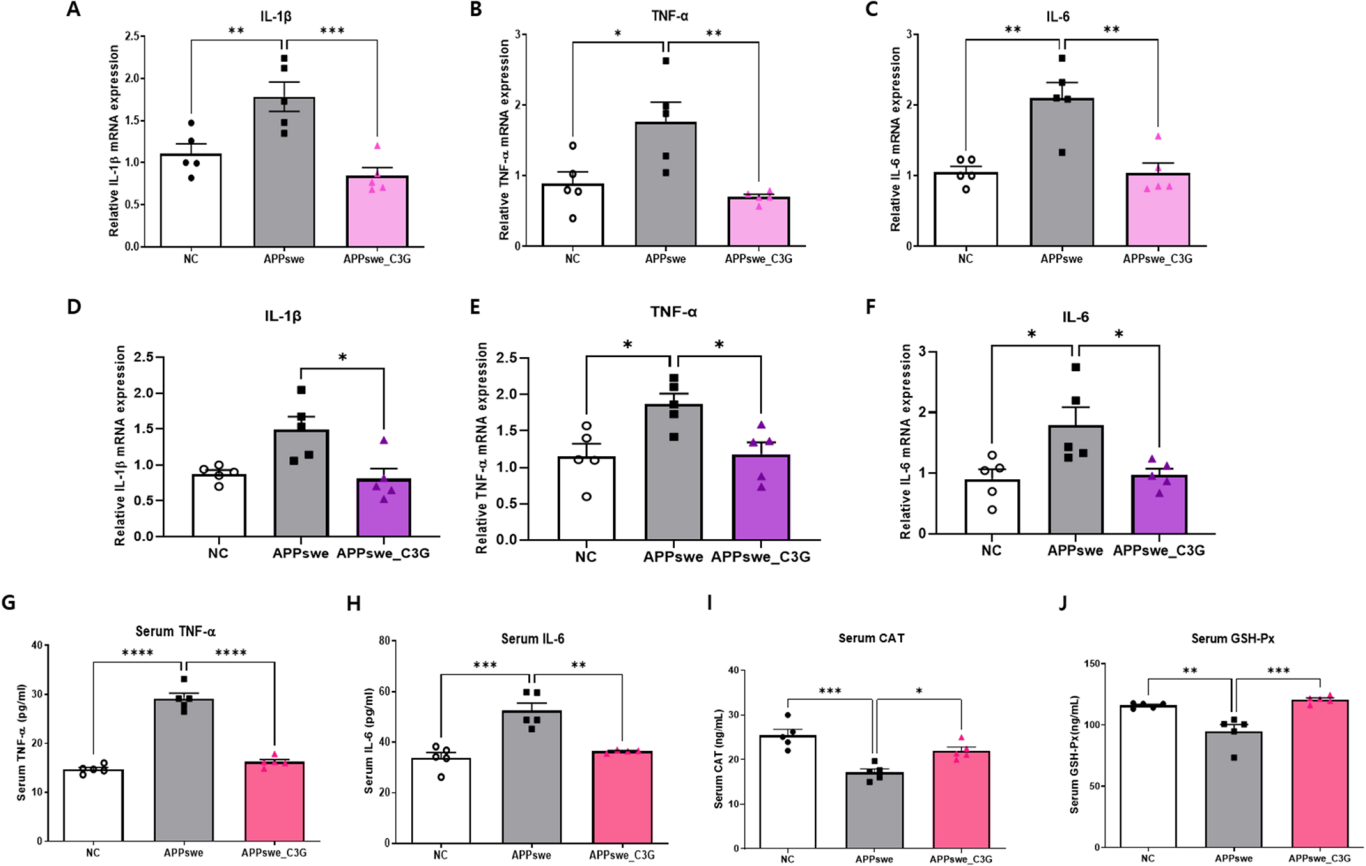
Cyanidin-3-O-glucoside (C3G) regulates neuroinflammation and antioxidation in APPswe mice. Mice were divided into three groups: non-transgenic mice (NC), AD mice orally administered PBS (APPswe), and AD mice orally administered 30 mg/kg/day C3G in PBS (APPswe_C3G). The mRNA expression levels of pro-inflammatory cytokines, such as interleukin (IL)-1β, tumor necrosis factor-α (TNF-α), and interleukin (IL)-6, in the cortical (**A**–**C**) and hippocampal regions of the brain (**D**–**F**) were observed using RT-PCR (n = 5). The levels of (**G**) TNF-α and (**H**) IL-6 and antioxidants (**I**) catalase (CAT) and (**J**) glutathione peroxidase (GSH-Px) were observed in the serum using ELISA (n = 5). Data are representative of three independent experiments with similar results. *p < 0.05, **p < 0.01, ***p < 0.001, ****p < 0.0001. Bars represent mean ± SE

Neuroinflammation and oxidative stress are two critical factors in the pathogenesis of NDs. IL-1β, TNF-α, and IL-6 are the three major pro-inflammatory cytokines released during the inflammatory processes [60]. The mRNA expression of these cytokines in the cortex and hippocampus and their protein expression in the serum were observed (Fig. 6). In the cortical and hippocampal regions of the APPswe_C3G mice, the mRNA expression of IL-1β (Fig. 6A and D), TNF- α (Fig. 6B and E), and IL-6 (Fig. 6C and F) was markedly downregulated compared to that in the APPswe group. The anti-inflammatory properties of C3G were apparent as the levels of pro-inflammatory cytokines were significantly downregulated in the APPswe_C3G group compared with those in the APPswe group. Similar to these results, the amount of pro-inflammatory cytokines TNF-α and IL-6 in the serum was significantly higher in the APPswe group than in the NC group and was lowered after C3G administration, as seen in the APPswe_C3G group (Figs. 6G, H). GSH-Px and CAT are antioxidants that protect tissues from damage and thus prevent tissues from oxidative stress [61]. In the APPswe group, both CAT and GSH-Px levels were significantly downregulated compared to those in the NC group but were significantly upregulated after C3G treatment (Fig. 6I, J).

### C3G attenuated neuronal apoptosis and tau protein phosphorylation in the cerebral cortex and hippocampus of APPswe mice

**Fig. 7:**
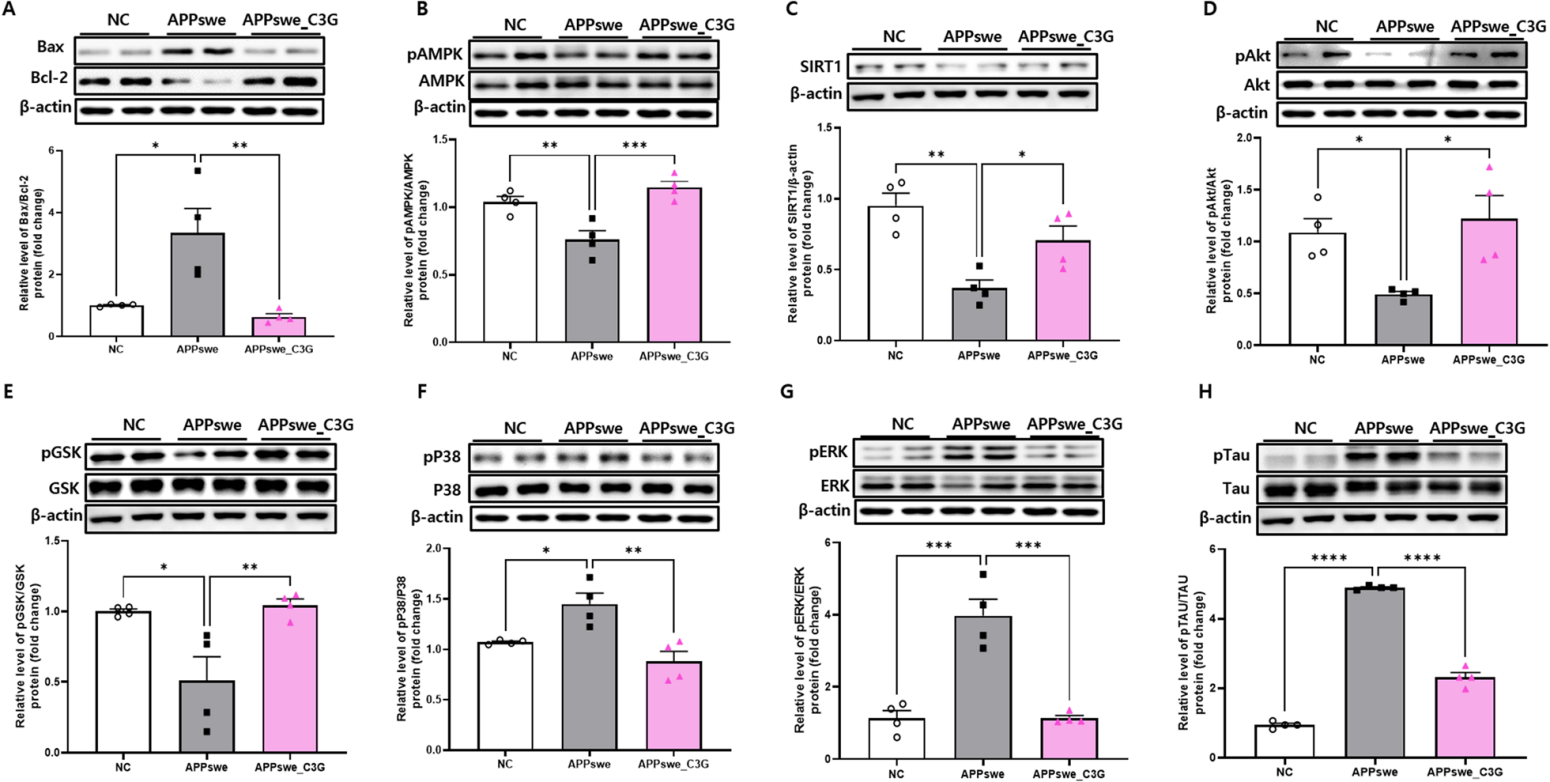
Cyanidin-3-O-glucoside (C3G) attenuated neuronal apoptosis and tau protein phosphorylation in the cortex of APPswe mice. Mice were divided into three groups: non-transgenic mice (NC), AD mice orally administered PBS (APPswe), and AD mice orally administered 30 mg/kg/day C3G in PBS (APPswe_C3G). Protein expression levels of different neuronal apoptosis-and tau protein phosphorylation-associated factors in the cortex were determined using sodium dodecyl sulfate–polyacrylamide gel electrophoresis (n = 4). Bcl-2-associated X protein (Bax)/B-cell lymphoma 2 (Bcl-2) (**A**); phospho-AMP-activated protein kinase (pAMPK)/AMPK (**B**); Sirtuin 1 (SIRT-1) (**C**); phospho-protein kinase B (Akt)/Akt (**D**); phospho- glycogen synthase kinase-3 beta (pGSK3β)/GSK3β (**E**); phospho-p38 MAPK (pP38)/p38 (**F**); phospho- extracellular signal-regulated kinases (pERK/ERK) (**G**), and phosphorylated tau (pTau/Tau) (**H**). Data are representative of three independent experiments with similar results. *p < 0.05, **p < 0.01, ***p < 0.001, ****p < 0.0001. Bars represent mean ± SE

**Fig. 8:**
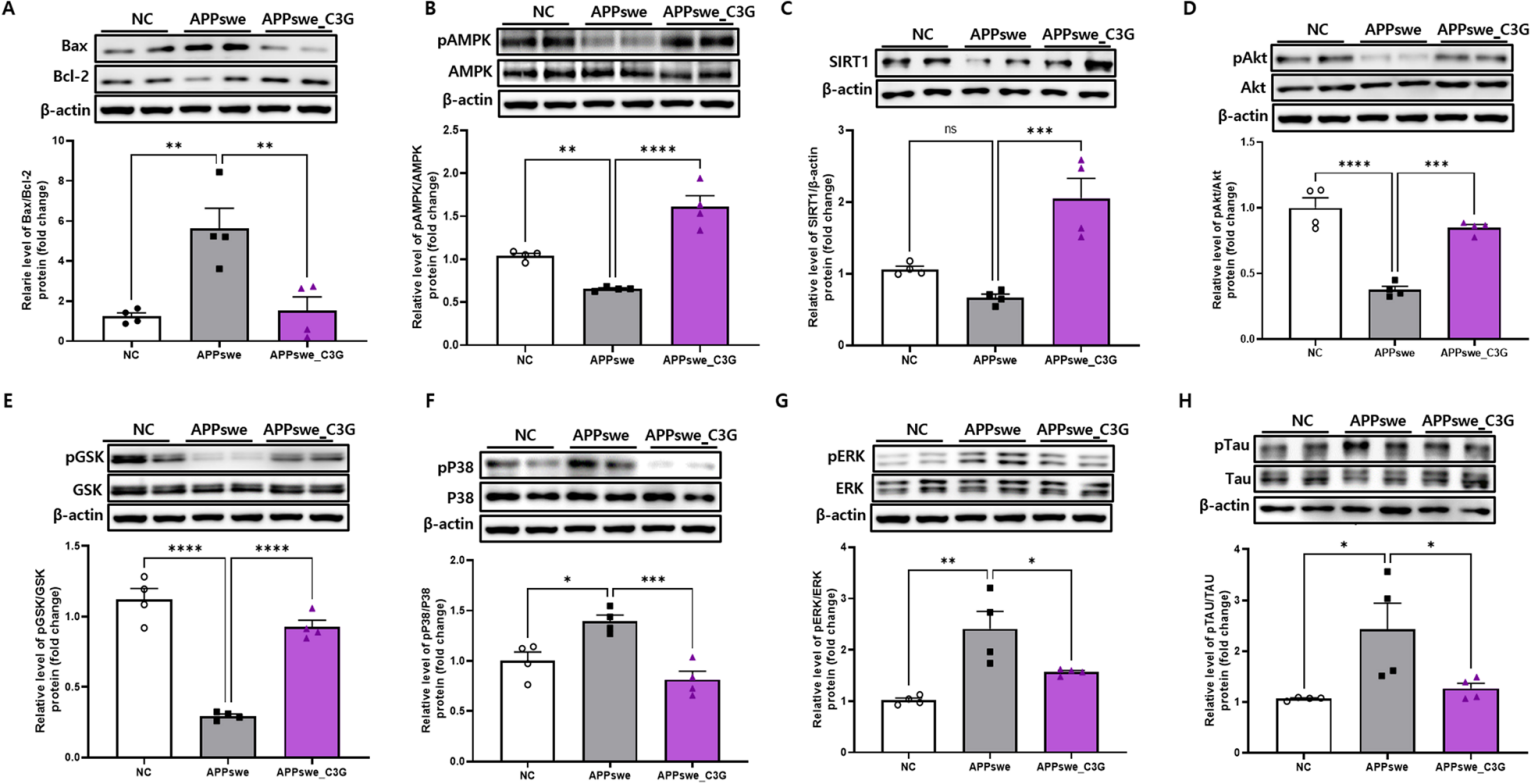
Cyanidin-3-O-glucoside (C3G) attenuated neuronal apoptosis and tau protein phosphorylation in the hippocampus of APPswe mice. Mice were divided into three groups: non-transgenic mice (NC), AD mice orally administered PBS (APPswe), and AD mice orally administered 30 mg/kg/day C3G in PBS (APPswe_C3G). The protein expression levels of different neuronal apoptosis- and tau protein phosphorylation-associated factors in the hippocampus were determined using western blotting (n = 4). Levels of Bcl-2-associated X protein (Bax)/B-cell lymphoma 2 (Bcl-2) (**A**); phospho-AMP-activated protein kinase (pAMPK)/AMPK (**B**); Sirtuin 1 (SIRT-1) (**C**); phospho-protein kinase B (Akt)/Akt (**D**); phospho- glycogen synthase kinase-3 beta (pGSK3β)/GSK3β (**E**); phospho-p38 MAPK (pP38)/p38 (**F**); phospho- extracellular signal-regulated kinases (pERK/ERK) (**G**); and phosphorylated tau (pTau/Tau) (**H**). Data are representative of three independent experiments with similar results. ns, not significant; *p < 0.05, **p < 0.01, ***p < 0.001, ****p < 0.0001. Bars represent mean ± SE

Neuronal apoptosis is a critical feature of NDs. The levels of apoptosis-related proteins were measured to determine the effects of C3G on neuronal apoptosis. Bax/Bcl2 ratio is a key marker for apoptosis [62]. APPswe mice showed significantly increased protein levels of Bax/Bcl2 compared to those in the NC group. However, C3G administration reversed this effect and significantly decreased Bax/Bcl2 levels compared to the APPswe group (Figs. 7A and 8A). In addition, inhibition of AMPK signaling has been reported as a marker of neuronal apoptosis in AD [63] and modulates tau phosphorylation and pathology in vivo [25]. SIRT1, a downstream signaling molecule of AMPK, plays a significant role in the pathogenesis of NDs. C3G treatment significantly upregulated the activation of AMPK (pAMPK/AMPK) and enhanced SIRT1 protein expression in the APPswe_C3G group compared with that in the APPswe group (Figs. 7B, C and 8B, C). Notably, the downregulation of SIRT1 protein expression was not significant in the cortical region of APPswe mice (Fig. 8C).

PI3K/Akt activation protects the cells from apoptosis [64]. Inhibition of PI3K/Akt can lead to the activation of pro-apoptotic markers, such as GSK3β. Further, activation of GSK3β induces post-translational modifications of the microtubule-associated protein tau and is also involved in tau protein hyperphosphorylation. However, phosphorylation of GSK3β inactivates its function [65]. In this study, the protein expression of both pAkt/Akt and pGSK3β/GSK3β were observed in the cortical and hippocampal regions of the brain. C3G administration significantly upregulated pAkt/Akt expression in APPswe_C3G mice and downregulated that in APPswe mice (Figs. 7D, E and 8D, E). Additionally, GSK3β activation was inhibited in the C3G-administered group; the pGSK3β/GSK3β level was significantly higher in the APPswe_C3G group than in the APPswe group.

MAPKs, such as P38 and ERK, are well-known indicators of cellular apoptosis, and their phosphorylation can result in their activation [23]. Recent studies have also reported a role for these MAPKs in tau hyperphosphorylation [24]. The protein levels of pP38/P38 and pERK/ERK were significantly higher in APPswe mice than in NC mice. In contrast, APPswe_C3G mice showed a significant reduction in the protein expression of pP38/P38 and pERK/ERK (Figs. 7F–G and 8F–G). This finding highlights the role of C3G in preventing neuronal apoptosis in AD. In addition, tau accumulation and phosphorylation need to be considered in the search for AD treatments. The pTau/Tau protein levels were significantly upregulated in the APPswe group compared to those in the NC group, while they were significantly downregulated in the APPswe_C3G group compared to those in the APPswe group (Figs. 7H and 8H). This finding could also be correlated with the reduced expression of phosphorylated MAPKs and increased expression of AMPK, pAkt/Akt, and pGSK3β/GSK3β. These results support the role of C3G in regulating tau protein phosphorylation.

### C3G enhanced synaptic plasticity in APPswe mice

**Fig. 9:**
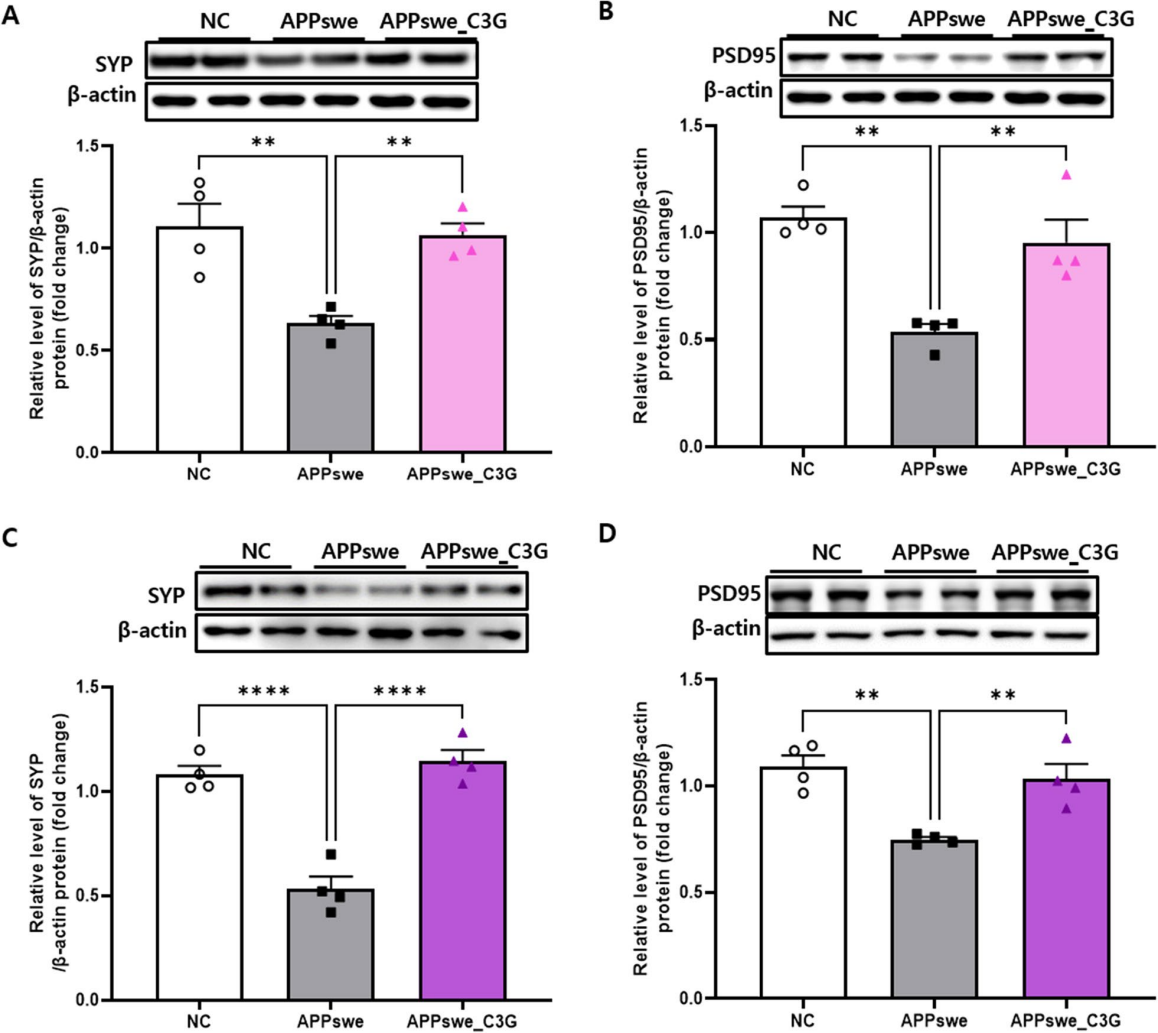
Cyanidin-3-O-glucoside (C3G) enhanced synaptic plasticity in the cortex and hippocampus APPswe mice. Mice were divided into three groups: non-transgenic mice (NC), AD mice orally administered PBS (APPswe), and AD mice orally administered 30 mg/kg/day C3G in PBS (APPswe_C3G). Data are mean ± SE **p < 0.01 and ****p < 0.0001. Expression levels of synaptic function-related proteins, such as synaptophysin (SYP) and postsynaptic density protein 95 (PSD95), were observed in the cortex (**A, B**) and hippocampus (**C, D**) using western blotting (n = 4). Data are representative of three independent experiments with similar results. **p < 0.01, ****p < 0.0001. Bars represent mean ± SE

Synaptic dysfunction is a major contributor to AD progression after Aβ plaques and NFTs [66]. After examining the significant effects of C3G on neuronal apoptosis and tau protein hyperphosphorylation, the role of C3G in synaptic dysfunction was investigated. The protein levels of SYP and PSD95 in the cortical and hippocampal regions of APPswe mice were significantly lower than those in NC mice. However, C3G demonstrated promising effects, as the APPswe_C3G group showed significantly higher protein levels of both SYP and PSD95 than the APPswe group (Fig. 9), indicating that C3G enhanced synaptic plasticity in APPswe mice.

## Discussion

Anthocyanins are promising therapeutic agents for treating NDs [67, 68]. C3G is one of the most common anthocyanins in black rice, black beans, purple potatoes, and many other colored berries. The bioactive potential of C3G is well-known as it exhibits various biological activities, such as antioxidant, anti-inflammatory, and anti-cancer effects. However, to the best of our knowledge, the potential of C3G in regulating the amyloidogenic pathway, autophagy, tau protein phosphorylation, neuronal cell death, and synaptic plasticity in an APPswe/PS1ΔE9 transgenic mouse model has yet to be reported.

The accumulation of Aβ plaques and NFTs are considered major hallmarks of AD. The soluble and insoluble Aβs (Aβ40 and Aβ42) are highly present in the cortical and hippocampal regions of the brain in AD disease pathology [6]. Zhao et al. found that treatment of flavone apigenin in APP/PS1 mice could reduce the cerebral levels of insoluble Aβ40 and Aβ42 significantly without affecting soluble Aβ levels [69]. Contrary to this, in our study, C3G was found to reduce both soluble and insoluble Aβ (Aβ40 and Aβ42) peptides in the cortical and hippocampal brain regions. The amyloidogenic pathway of AD shows improper cleavage of APP through BACE1, which produces insoluble Aβ peptides that accumulate in the brain to induce toxicity [5]. In the previously reported studies, quercetin and apigenin were found to be effective in ameliorating cognitive functioning in APP/PS1 mice and reduced CTFβ and BACE1 [69, 70]. Our study observed that C3G administration to APPswe mice reduced the protein expression levels of APP, PS1, and BACE1, showing C3G as a potential therapeutic agent in targeting the amyloidogenic pathway of AD. Song et al. demonstrated that C3G administration could improve cognitive behavior in APPswe/PS1ΔE9 mouse model [71]. Likewise, C3G enhanced cognitive learning.

Autophagy is another important aspect of AD, and aberrant autophagic functions in neurons of AD models have been previously reported [13, 14]. Enhanced Beclin-1 and LC3 and reduced SQSTM1/p62 expression by β-asarone attenuated Aβ40 and Aβ42 levels in an in vivo AD model [72]. Similar to previous findings, we observed increased LC3 expression and decreased SQSTM1/p62 expression in the C3G-administered group, which might be attributed to the clearance of Aβ40 and Aβ42 levels in APPswe mice following C3G treatment. The increased expression of the autophagy markers LC3B-II, LAMP1, and TFEB is also involved in inducing autophagy flux and lysosomal biogenesis upon celastrol (a compound isolated from *Tripterygium wilfordii*) application [73]. In our study, C3G-treated APPswe mice showed upregulated expression of LC3B-II, LAMP-1, and TFEB in the cortical and hippocampal regions of the brain compared to APPswe mice. PPARα activation [58] and TFEB expression were also found to play an important role in autophagy [74]. We found increased PPARα protein expression upon C3G administration, indicating the significant role of PPARα in autophagy.

Chronic activation of the microglia due to the presence of Aβ plaques and NFTs could activate the pro-inflammatory cascade, which can damage other cells of the central nervous system [75]. Nobiletin was found to attenuate neuroinflammation in APP/PS1 mice by downregulating protein levels of IL-1β, TNF-α, and IL-18 [76]. Similarly, our study found that C3G reduced mRNA expression levels of common pro-inflammatory cytokines such as IL-1β, TNF-α, and IL-6 and protein expression levels in APPswe mice.

Oxidative stress plays a major role in AD disease progression [77]. A previous study reported by Sukprasansap et al. demonstrated the antioxidative potential of C3G in activating the expression of endogenous antioxidant enzymes such as superoxide dismutase (SOD), CAT, and GSH-Px [78]. Following the previous findings, our study also found that C3G could be seen as a potential antioxidant as it upregulated the expression of GSH-Px and CAT. Neuronal apoptosis or cell death is a characteristic feature of neurodegeneration. Different signaling molecules (Bax/Bcl2) and pathways (PI3K/Akt and MAPK) affect neuronal cell death and tau hyperphosphorylation. The relative ratio of the pro-apoptotic protein Bax to the anti-apoptotic protein Bcl-2 is important for regulating the release of caspase activity-inducing factors from the mitochondria and has been linked to various pathological conditions characterized by cell death [79]. A previous study reported that apigenin ameliorated oxidative stress-induced neuronal cell death in SH-SY5Y cells by downregulating the Bax/Bcl2 ratio [80]. Similarly, in this study, APPswe mice showed upregulated Bax/Bcl2 ratio in the cortical and hippocampal regions of the brain, indicating neuronal caspase-induced neuronal apoptosis. However, C3G administration inhibited neuronal apoptosis by upregulating Bcl2 expression and downregulating the Bax/Bcl2 ratio.

AMPK has protective and reparative effects on developing and mature neurons. AMPK is activated in response to stresses that deplete the cellular ATP supply, such as low glucose levels, hypoxia, ischemia, and heat shock, in response to an increase in the AMP/ATP ratio [81]. AMPK downregulation is linked with tau pathology and memory impairment, and AMPK-SIRT1 pathway dysfunction contributes to neuronal apoptosis and cognitive impairment [82, 83]. AMPK is negatively correlated with p38 MAPK expression, and overexpression of AMPK can inhibit the expression of p38, weakening p38-induced pro-apoptosis [84]. Similar to these studies, our results showed that C3G administration increased AMPK and SIRT1 expression and pP38/P38 deactivation.

Impaired PI3K/Akt/GSK-3β signaling has been investigated in the brains of patients with AD and AD mouse models. Ali et al. reported that natural dietary supplementation with anthocyanins alleviated oxidative stress, neurodegeneration, and memory impairment in an in vivo AD model [85]. Another study group reported the neuroprotective effects of the natural flavonoid wogonin in regulating tau protein phosphorylation in SH-SY5Y cells by inhibiting GSK3β [86]. Likewise, we observed that C3G supplementation re-activated pAkt/Akt and deactivated GSK3β. Moreover, as observed through pTau/Tau expression, tau protein phosphorylation was upregulated in APPswe mice. However, pTau/Tau expression was downregulated in the APPswe_C3G group. These results indicated the neuroprotective role of C3G in mediating different signaling pathways that lead to tau protein phosphorylation and neuronal apoptosis.

Synaptic loss and dysfunction are key features of many neurodegenerative diseases, including AD [66]. Liu et al. showed that inhalation of lemon essential oil improved cognitive dysfunction in APPswe/PS1ΔE9 mice by enhancing the synapse-associated proteins, PSD95 and SYN, and brain-derived neurotrophic factor and reducing acetylcholinesterase levels [87]. Corroborating previous findings, we found that the downregulated expression of PSD95 and SYN in both the cortical and hippocampal regions of APPswe mouse brains was restored in the APPswe_C3G group, indicating that C3G enhanced synaptic function and plasticity.

Our study confirmed various brain-protective effects of C3G using an AD mice model expressing a chimeric mouse/human amyloid precursor protein associated with AD. There are many restrictions on applying this directly to humans. Nevertheless, after 16 weeks of oral administration, both Aβ1-40 and Aβ1-42 levels were significantly reduced by C3G (30 mg/kg/day). In addition, considering the behavioral improvement in the Y-maze test, the practical brain protection effect of C3G is likely to be possible in all animals, including humans.

## Conclusions

In conclusion, C3G could alleviate AD pathology by improving cognitive functioning, clearing Aβ plaques, reducing inflammation and enhancing antioxidation, attenuating tau protein phosphorylation and neuronal apoptosis, and enhancing autophagy and neuronal plasticity.

## Data Availability

All of the data is contained within the article.
